# Detection of West Nile virus lineage 2 in North‐Eastern Spain (Catalonia)

**DOI:** 10.1111/tbed.13086

**Published:** 2018-12-26

**Authors:** Núria Busquets, Minerva Laranjo‐González, Mercè Soler, Olga Nicolás, Raquel Rivas, Sandra Talavera, Rubén Villalba, Elena San Miguel, Núria Torner, Carles Aranda, Sebastian Napp

**Affiliations:** ^1^ IRTA, Centre de Recerca en Sanitat Animal (CReSA IRTA‐UAB) Bellaterra Spain; ^2^ Departament d'Agricultura, Ramaderia, Pesca i Alimentació Generalitat de Catalunya Servei de Prevenció en Salut Animal Barcelona Spain; ^3^ Departament de Territori i Sostenibilitat Centre de Fauna de Vallcalent Lleida Spain; ^4^ Laboratorio Central de Veterinaria Ministerio de Agricultura y Pesca, Alimentación y Medio Ambiente (MAPAMA) Madrid Spain; ^5^ Public Health Agency of Catalonia Barcelona Spain; ^6^ CIBER Epidemiology and Public Health (CIBERESP) Madrid Spain; ^7^ Servei de Control de Mosquits Consell Comarcal del Baix Llobregat Sant Feliu de Llobregat Spain

## Abstract

In September 2017, West Nile virus (WNV) lineage 2 was detected in Catalonia (Northern Spain) in northern goshawks by passive surveillance. The phylogenetic analyses showed that it was related to the Central/Southern European strains, evidencing WNV lineage 2 spread to Western Europe. WNV local transmission was later detected in bearded vultures housed at the Wildlife Recovery center where the goshawk was transferred to. Further studies, before the following period of high mosquito activity, indicated that WNV had circulated intensively in poultry and horses but only surrounding of the area where the virus was detected. In other areas of Catalonia, circulation of flaviviruses different to WNV was identified. Public Health investigations failed to detect WNV infection in humans.

## 1 INTRODUCTION

West Nile virus (WNV), within the genus *Flavivirus*, family *Flaviviridae*, is the most widespread arbovirus. Its transmission cycle involves mosquitoes, mainly *Culex* spp. and birds. Horses and humans are considered dead‐end hosts, and their infection is usually asymptomatic or mild, but sporadically severe disease with neurological symptoms or even death may occur.

West Nile virus has circulated in Europe for decades, but up until 2004, all outbreaks had been caused by WNV lineage 1. However, in 2004, WNV lineage 2 was detected in Hungary in a northern goshawk (*Accipiter gentilis*) with neurological symptoms, the first report of this lineage outside Africa (Bakonyi et al., 2006). In the following years, WNV lineage 2 spread within Eastern and Central/Southern Europe, where the virus has remained endemic, causing hundreds cases in humans, while cases in horses have been less frequently reported (Hernández‐Triana et al., 2014; Napp, Petric, & Busquets, 2018).

In Spain, cases of WNV in horses and humans were reported in 2010 in the South of the country (Andalusia region), which were caused by a WNV lineage 1 strain (García‐Bocanegra et al., 2011). Since then, WNV became endemic in Southern Spain, re‐emerging every year and expanding northwards, causing outbreaks in horses, with further confirmed human cases in 2016 (López‐Ruiz et al., 2018).

## 2 RESULTS AND DISCUSSION

In September 2017, a northern goshawk (*Accipiter gentilis*) was found with dehydration, apathy and low weight near an urban area of Lleida province (Catalonia, Spain), and was transferred to the Wildlife Recovery Center (WRC) of Vallcalent. Five days later it developed nervous symptoms (head‐shaking, incoordination, and inability to stand upright), and was euthanized. Samples of nervous tissue were positive to WNV infection by RT‐qPCR (Linke, Ellerbrok, Niedrig, Nitsche, & Pauli, 2007). WNV‐positivity was confirmed by the Central Veterinary Laboratory (CVL) in Algete by RT‐qPCR (Jiménez‐Clavero, Agüero, Rojo, & Gómez‐Tejedor, 2006). The analysis of a partial sequence of the *NS5* gene using the primers described by Scaramozzino et al., 2001; indicated that it belonged to lineage 2. After confirmation, the case was reported to the World Organization for Animal Health (OIE). Within days, WNV lineage 2 was also detected in another sick northern goshawk that was admitted to Vallcalent‐WRC and had died after 2 days. Three days later, a third northern goshawk found with an old fracture tested positive by cELISA (IDvet‐ID Screen^®^ West Nile Competition) and was confirmed by SNT at the CVL (titer of 1/40).

West Nile virus infection was detected in three northern goshawks, including two symptomatic animals, even though, according to the ornithologists consulted, this species is uncommon in the affected area, and probably just arrived (autumn migration) from Northern‐Eastern Europe. Interestingly, in Europe, WNV lineage 2 has been repeatedly isolated from northern goshawks (Bakonyi et al., 2013). This may be explained by a higher susceptibility of this species, and by the fact that goshawks feed mainly on birds (García‐Salgado et al., 2015), which if infected may result in a subsequent infection of the predator (Komar et al., 2003). In any case, northern goshawks should be considered a key species in WNV surveillance in Europe.

By the same time, one bearded vulture (*Gypaetus barbatus*), a near threatened‐species housed in Vallcalent‐WRC, showed neurological symptoms compatible with WNV infection. The animal tested positive by cELISA and negative by SNT, but 1‐month later antibodies against WNV were detected by SNT, evidencing seroconversion, and WNV transmission within Vallcalent‐WRC. Serum samples from other 13 bearded vultures were positive by cELISA, and two were confirmed by SNT at the CVL (titers between 1/20 and 1/40).

**Figure 1:**
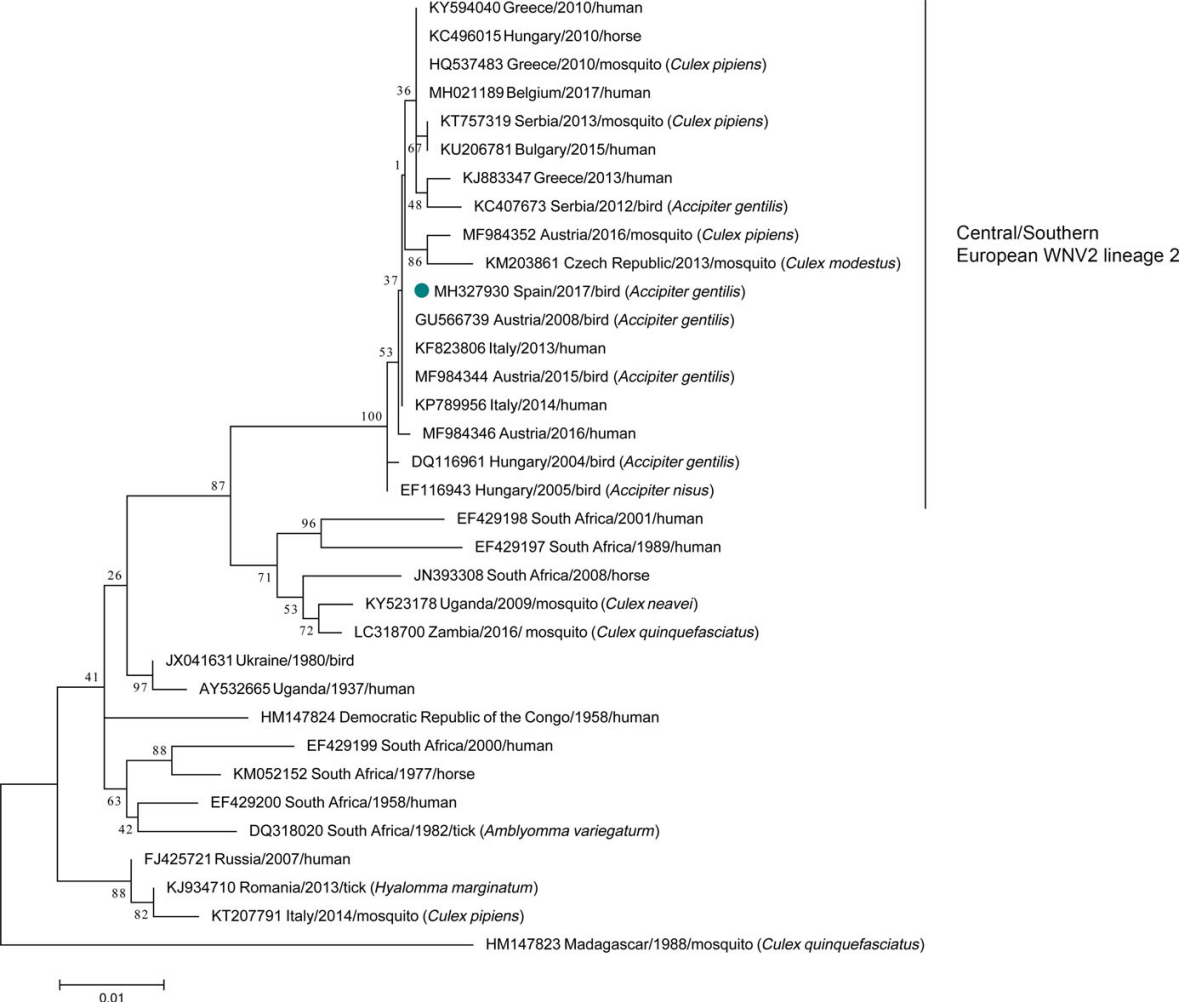
Molecular phylogenetic analysis by maximum likelihood method. The evolutionary history was inferred by using the Maximum Likelihood method based on the Tamura‐Nei model (Tamura & Nei, 1993). The tree with the highest log likelihood (−2,521.85) is shown. The percentage of trees in which the associated taxa clustered together is shown next to the branches. Initial tree(s) for the heuristic search were obtained automatically by applying Neighbor‐Join and BioNJ algorithms to a matrix of pairwise distances estimated using the Maximum Composite Likelihood (MCL) approach, and then selecting the topology with superior log likelihood value. The tree is drawn to scale, with branch lengths measured in the number of substitutions per site. The analysis involved 34 nucleotide sequences. Codon positions included were 1st + 2nd + 3rd + Noncoding. All positions containing gaps and missing data were eliminated. There were a total of 933 positions in the final dataset. Evolutionary analyses were conducted in MEGA7 (Kumar, Stecher, & Tamura, 2016). The Spanish WNV isolate is marked using a dot. The sequence obtained in this study was submitted to GenBank Nucleotide Sequence Database under the following accession number: MH327930. The Spanish WNV isolate is marked using a green dot. The sequence obtained in this study [Colour figure can be viewed at wileyonlinelibrary.com]

A fragment of 930 nt of the *NS5* gene from the first northern goshawk was sequenced by Sanger's method using the primers described by Vázquez et al. (2012) after WNV isolation in Vero cells. Comparisons with published sequences were performed by searches in the NCBI BLAST database, and sequences were aligned by Clustal W. Phylogenetic analyses showed that the WNV lineage 2 isolated in Spain belonged to the Central/Southern European WNV lineage 2 cluster (Ravagnan et al., 2015), where WNV strains detected in the last years in other northern goshawks, humans, and mosquitoes are included (Figure 1).

The detection in the northern goshawk was the first WNV outbreak reported in the North of the Iberian Peninsula. Furthermore, until now, all WNV cases reported there had been caused by WNV lineage 1. Our results evidence the westward spread of WNV lineage 2 in Europe, a strain that so far had been restricted to the Central/South and Eastern areas of the continent.

Detection of WNV in birds by molecular techniques implied the activation of the Emergency Program for WNV in Catalonia. Public Health authorities and people in contact with horses (i.e., veterinarians and owners) in the area were informed. Vector surveillance was conducted in Vallcalent‐WRC at the end of October using three BG traps baited with CO_2_, but no mosquitoes were trapped, as temperature had dropped by that time. However, several potential mosquito breeding sites were observed in the center, and high biting mosquito activity had been reported by members of the WRC staff during the previous summer (personal communication).

**Figure 2:**
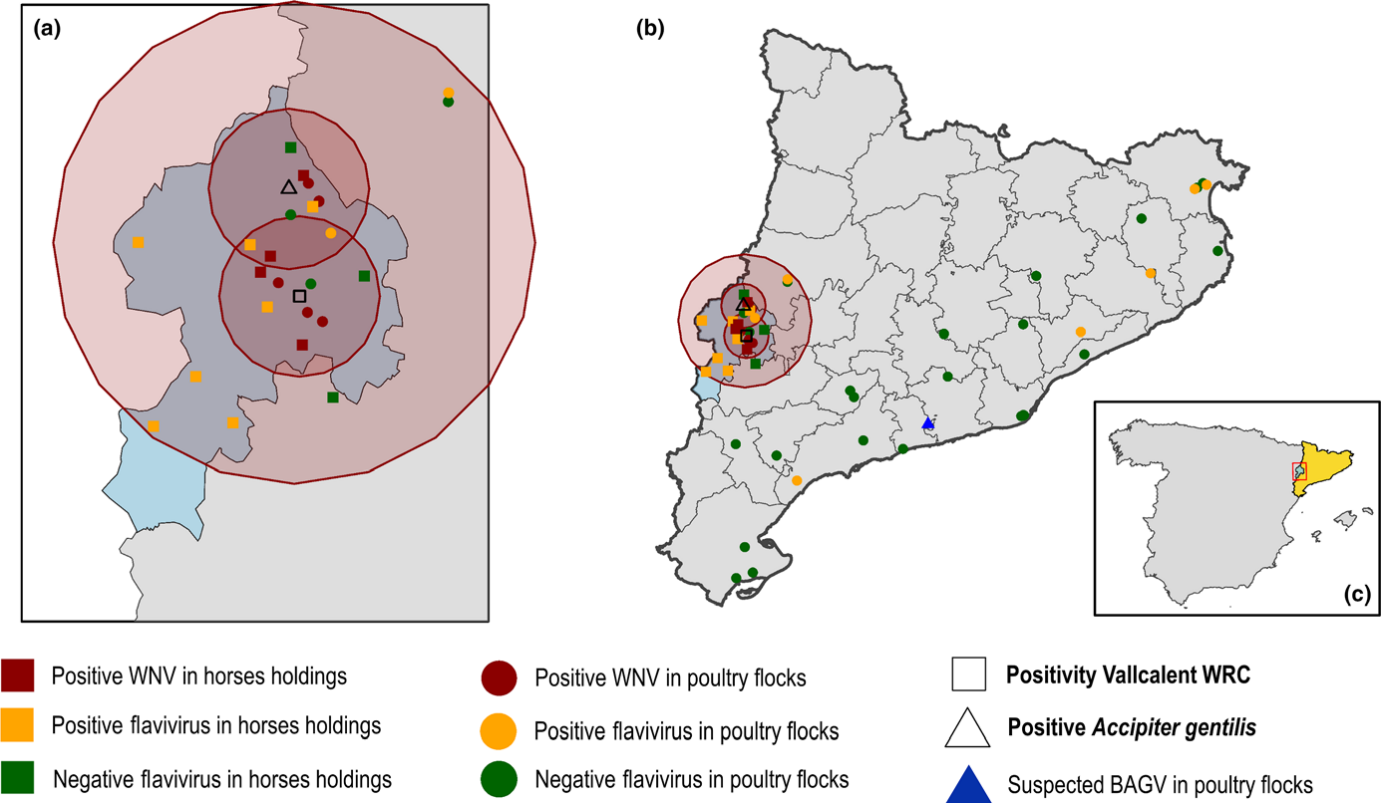
(a) Locations where the WNV‐positive goshawk was detected (triangle), and of Vallcalent‐WRC (empty square). Also results of the serological cross‐sectional survey in poultry (round symbol) and horses (square symbol) carried out in the surveillance area (10 km radius). (b) Results of the cross‐sectional survey in poultry (round symbol) and horses (square symbol) carried out in the surveillance area (10 km radius). (c) Location of Catalonia, and the affected area, within Spain [Colour figure can be viewed at wileyonlinelibrary.com]

To evaluate the extent of WNV circulation, in October 2017, a surveillance zone of 10 km around the first positive goshawk and Vallcalent‐WRC was defined, and within that area, a cross‐sectional survey in poultry and horses was carried out. Chickens and horses were first tested by cELISA, and positive samples were confirmed by SNT at the CVL. The SNT performed included Bagaza virus (BAGV) and WNV in the virus panel, but no other flaviviruses such as Usutu virus (USUV) which was detected in another area of Catalonia in 2006 (Busquets, Alba, Allepuz, Aranda, & Ignacio, 2008) and could cause cross‐reactivity. The results indicated a potential widespread WNV circulation within the surveillance zone in both chickens and horses (Table 1 and Figure 2a & c). In addition, one of the horses positive by cELISA gave a doubtful result by IgM ELISA (INgezim West Nile IgM, Ingenasa), but was negative by SNT. No symptoms compatible with WNV were observed in any of the animals sampled. Given positivity in the surrounding area, a second cross‐sectional survey was designed to further evaluate the extent of WNV circulation by serological techniques. The survey was carried out in 2018, before the period of high vector activity. In horses, the area was extended to a radius of 30 km around the midpoint between Vallcalent‐WRC, while in poultry, the second survey covered the whole of Catalonia. Of the seven horses’ holdings analyzed, one further positive to WNV (although within the initial 10 km radius), and four positives for flaviviruses, were detected (Table 1 and Figure 2b). In poultry, of the 29 flocks evaluated (Table 1 and Figure 2b), seven were positive for flaviviruses, and in one flock one bird showed higher antibody titers against Bagaza virus than for WNV by SNT. Therefore, an intense WNV circulation in the surroundings (i.e., 10 km radius) of VRC and where the first positive goshawk was found, but no positivity beyond that indicating either low‐level or no circulation of WNV in the rest of Catalonia. The results also indicated circulation of other flaviviruses, which seemed very intense in the Segrià area as evidenced in horses, but which also occurred in other areas as evidenced in poultry, although it did seem less intense. There seemed to be BAGV circulation in a chicken holding in Central‐Western Catalonia. The identity of the other flavivirus/flaviviruses involved, as well as their epidemiological relevance for horses, birds or other animals, including humans, should be investigated.

**Table 1:** Results of the cross‐sectional survey in chickens and horses within the surveillance area. Results of cELISA and SNT for both individual animals and holdings

	Animals			Holdings		
	Tested	Positive cELISA	Positive SNT (titers)	Tested	Positive cELISA	Positive SNT
First survey						
Chickens	308	46	32 (1/10–1/80)	8	6	5
Horses	119	21	6 (all 1/10)	7	6	3
Second survey						
Chickens	788	10	2^a^ (1/80 & 1/640)	29	7	1^a^
Horses	19	10	3 (1/10–1/40)	7	5	1

a Animal 1/640 positive to WNV was also positive to BAGV (1/1,260), while the serum of the 1/80 positive to WNV resulted citotoxic when BAGV was tested.

Even though WNV lineage 2 has caused hundreds of human cases in Eastern and Central/Southern Europe (Napp et al., 2018), the epidemiological investigation carried out in Catalonia by the Public Health authorities, indicated no increase in cases of non‐specific viral meningitis or encephalitis in humans between May and October 2017 in the province affected. In addition, 211 blood samples from donors collected in that province were tested for WNV IgG and were all negative (Blood and Tissue bank, personal communication). That seems to suggest that there was no widespread circulation of WNV in humans in the area.

The fact that WNV was detected in Catalonia was probably due to the implementation of a comprehensive surveillance program (including active and passive surveillance in wild birds and horses) for more than 12 years. In fact, since 2010, WNV positivity by SNT has been detected in wild birds (mainly raptors) in this same area, which was not included among the high‐risk areas for WNV in Catalonia (Alba et al., 2014). However, local WNV circulation could never be demonstrated as positive animals were always potentially migratory.

Given the ability of WNV to overwinter (Rudolf et al., 2017), the re‐emergence of WNV lineage 2 in Catalonia would be possible. After the detection of WNV circulation in 2017, surveillance measures implemented in the affected area were intensified.

Furthermore, WNV lineage 2 recurrence combined with the northward expansion of WNV lineage 1, the emergence of novel WNV genotypes and the circulation of other flaviviruses, e.g., USUV (Busquets et al., 2008) results in a complex scenario for the future, with several co‐circulating flaviviruses. The consequences of flaviviruses co‐infections in both the hosts and the vectors are unpredictable (Rizzoli et al., 2015).

## References

[tbed13086-bib-0001] Alba, A. , Allepuz, A. , Napp, S. , Soler, M. , Selga, I. , Aranda, C. , … Busquets, N. (2014). Ecological surveillance for West Nile in Catalonia (Spain), learning from a five‐year period of follow‐up. Zoonoses Public Health, 61(3), 181–191. 10.1111/zph.12048 23590452

[tbed13086-bib-0002] Bakonyi, T. , Ferenczi, E. , Erdélyi, K. , Kutasi, O. , Csörgő, T. , Seidel, B. , … Nowotny, N. (2013). Explosive spread of a neuroinvasive lineage 2 West Nile virus in Central Europe, 2008/2009. Veterinary Microbiology, 165(1–2), 61–70. 10.1016/j.vetmic.2013.03.005 23570864

[tbed13086-bib-0003] Bakonyi, T. , Ivanics, E. , Erdélyi, K. , Ursu, K. , Ferenczi, E. , Weissenböck, H. , & Nowotny, N. (2006). Lineage 1 and 2 strains of encephalitic West Nile virus, central Europe. Emerging Infectious Diseases, 12(4), 618–623. 10.3201/eid1204.051379 16704810 PMC3294705

[tbed13086-bib-0004] Busquets, N. , Alba, A. , Allepuz, A. , Aranda, C. , & Ignacio, N. J. (2008). Usutu virus sequences in *Culex pipiens* (Diptera: Culicidae), Spain. Emerging Infectious Diseases, 14(5), 861–863. 10.3201/eid1405.071577 18439389 PMC2600269

[tbed13086-bib-0005] García‐Bocanegra, I. , Jaén‐Téllez, J. A. , Napp, S. , Arenas‐Montes, A. , Fernández‐Morente, M. , Fernández‐Molera, V. , & Arenas, A. (2011). West Nile fever outbreak in horses and humans, Spain. Emerging Infectious Diseases, 17(12), 2397–2399. 10.3201/eid1712.110651 22172565 PMC3311180

[tbed13086-bib-0006] García‐Salgado, G. , Rebollo, S. , Pérez‐Camacho, L. , Martínez‐Hesterkamp, S. , Navarro, A. , & Fernández‐Pereira, J. M. (2015). Evaluation of trail‐cameras for analyzing the diet of nesting raptors using the Northern Goshawk as a model. PLoS ONE, 10(5), e0127585 10.1371/journal.pone.0127585 25992956 PMC4438871

[tbed13086-bib-0007] Hernández‐Triana, L. M. , Jeffries, C. L. , Mansfield, K. L. , Carnell, G. , Fooks, A. R. , & Johnson, N. (2014). Emergence of west nile virus lineage 2 in europe: A review on the introduction and spread of a mosquito‐borne disease. Frontiers in Public Health, 2, 271 10.3389/fpubh.2014.00271 25538937 PMC4258884

[tbed13086-bib-0008] Jiménez‐Clavero, M. A. , Agüero, M. , Rojo, G. , & Gómez‐Tejedor, C. (2006). A new fluorogenic real‐time RT‐PCR assay for detection of lineage 1 and lineage 2 West Nile viruses. Journal of Veterinary Diagnostic Investigation, 18(5), 459–462. 10.1177/104063870601800505 17037613

[tbed13086-bib-0009] Komar, N. , Langevin, S. , Hinten, S. , Nemeth, N. , Edwards, E. , Hettler, D. , … Bunning, M. (2003). Experimental infection of North American birds with the New York 1999 strain of West Nile virus. Emerging Infectious Diseases, 9(3), 311–312. 10.3201/eid0903.020628 12643825 PMC2958552

[tbed13086-bib-0010] Kumar, S. , Stecher, G. , & Tamura, K. (2016). MEGA7: Molecular evolutionary genetics analysis version 7.0 for bigger datasets. Molecular Biology and Evolution, 33(7), 1870–1874. 10.1093/molbev/msw054 27004904 PMC8210823

[tbed13086-bib-0011] Linke, S. , Ellerbrok, H. , Niedrig, M. , Nitsche, A. , & Pauli, G. (2007). Detection of West Nile virus lineages 1 and 2 by real‐time PCR. Journal of Virological Methods, 146(1–2), 355–358. 10.1016/j.jviromet.2007.05.021 17604132

[tbed13086-bib-0012] López‐Ruiz, N. , Montaño‐Remacha, M. D. C. , Durán‐Pla, E. , Pérez‐Ruiz, M. , Navarro‐Marí, J.M. , Salamanca‐Rivera, C. , … Ruiz‐Fernández, J. (2018). West Nile virus outbreak in humans and epidemiological surveillance, west Andalusia, Spain, 2016. Eurosurveillance, 23(14):17–00261. 10.2807/1560-7917.es.2018.23.14.17-00261 PMC5894251 29637890

[tbed13086-bib-0013] Napp, S. , Petric, D. , & Busquets, N. (2018). West Nile virus and other mosquito‐borne viruses present in Eastern Europe. Pathogens and Global Health, 112, 1–233–248. 10.1080/20477724.2018.1483567 29979950 PMC6225508

[tbed13086-bib-0014] Ravagnan, S. , Montarsi, F. , Cazzin, S. , Porcellato, E. , Russo, F. , Palei, M. , … Capelli, G. (2015). First report outside Eastern Europe of West Nile virus lineage 2 related to the Volgograd 2007 strain, northeastern Italy, 2014. Parasites & Vectors, 8, 418 10.1186/s13071-015-1031-y 26265490 PMC4534017

[tbed13086-bib-0015] Rizzoli, A. , Jimenez‐Clavero, M. A. , Barzon, L. , Cordioli, P. , Figuerola, J. , Koraka, P. , … Tenorio, A. (2015). The challenge of West Nile virus in Europe: Knowledge gaps and research priorities. Eurosurveillance, 20(20). pii: 21135. 10.2807/1560-7917.es2015.20.20.21135 26027485

[tbed13086-bib-0016] Rudolf, I. , Betášová, L. , Blažejová, H. , Venclíková, K. , Straková, P. , Šebesta, O. , … Hubálek, Z. (2017). West Nile virus in overwintering mosquitoes, central Europe. Parasites & Vectors, 10(1), 452 10.1186/s13071-017-2399-7 28969685 PMC5625652

[tbed13086-bib-0017] Scaramozzino, N. , Crance, J. M. , Jouan, A. , DeBriel, D. A. , Stoll, F. , & Garin, D. (2001). Comparison of flavivirus universal primer pairs and development of a rapid, highly sensitive heminested reverse transcription‐PCR assay for detection of flaviviruses targeted to a conserved region of the NS5 gene sequences. Journal of Clinical Microbiology, 39(5), 1922–1927. 10.1128/JCM.39.5.1922-1927.2001 11326014 PMC88049

[tbed13086-bib-0018] Tamura, K. , & Nei, M. (1993). Estimation of the number of nucleotide substitutions in the control region of mitochondrial DNA in humans and chimpanzees. Molecular Biology and Evolution, 10(3), 512–516. 8336541 10.1093/oxfordjournals.molbev.a040023

[tbed13086-bib-0019] Vázquez, A. , Sánchez‐Seco, M. P. , Palacios, G. , Molero, F. , Reyes, N. , Ruiz, S. , … Tenorio, A. (2012). Novel flaviviruses detected in different species of mosquitoes in Spain. Vector‐Borne and Zoonotic Diseases, 12(3), 223–229. 10.1089/vbz.2011.0687 22022811 PMC3300060

